# The Function of Lactation and Artificial Feeding

**Published:** 1913-02-08

**Authors:** 


### MATERNITY AND MOTHERING.

(Inquiries and Correspondence are invited.)

### The Function of Lactation and Artificial Feeding.

Every book on the care of infants, whether scientific or popular in scope, rightly insists nowadays upon the paramount advantage to a baby of maternal nursing, and upon the duty which a mother owes to her child to give it this advantage if it be within her power. Unfortunately, as is well known, there are many causes which militate against this ideal. Among the poor the employment of mothers in factories and similar occupations which entail absence from home are common reasons for artificial feeding. Among the rich luxury and its attendant selfishness produce the same result only too often. But in both classes cases are met with in which, with the most earnest desire to nurse their babies, mothers find themselves with little or no power to do so satisfactorily.

Sometimes, though rarely, lactation is never properly established at all, and hand-feeding is necessary right from the start; this occurs in quite healthy women, whom one would expect to have no difficulty of the sort. More often the lacteal secretion is normally produced, but owing to some physical malformation of the nipple it is impossible for the infant to procure it in the ordinary way. With patience and perseverance and the use of a breast pump this difficulty can generally be surmounted, but there are cases in which it proves so intractable that nursing has to be abandoned. More commonly still lactation is at first performed normally and satisfactorily, or nearly so, but fails some time or other during the first three months. Often there is no apparent cause for this, but in a good many cases the diminution of secretion takes place when the mother first begins to get about, two or three weeks after confinement.

### Substitutes for Breast Feeding.

Granted, then, that to suckle her infant for the normal eight or nine months is not within the power of every mother, it becomes an important matter to ascertain the frequency of this impotency and to know what steps can be taken to minimise its likelihood. The most exhaustive researches into this matter have been carried out in the United States, and they are full of interest to all who may have to supervise or to conduct the lofty function of maternal nursing. It has to be borne in mind that the population of America is much more heterogeneous than that of Great Britain; that it includes nationalities and race-strains practically unknown over here; and that conditions of life are in many ways rather different in the two countries. Therefore some slight caution is necessary in applying to British parents statistics and conclusions drawn from American sources. But in the main it cannot be doubted that what is true of citizens of the United States is true in Great Britain, as far as questions such as those now being discussed are concerned.

Dr. Jacobi, easily one of the most experienced and respected of the leaders of American medical science, pronounced only last June that "there is no such thing as absolute absence of milk secretion." That may in strictness be the case, but for practical purposes it is too sweeping, since cases of milk secretion so poor in quality and quantity as to be of no value do certainly exist. Schwarz at Baltimore was able by careful attention to, and education of, mothers attending the hospital to get 96 per cent. of babies breast-fed for one month, 88 per cent. for three months, and 77 per cent. for six months. His cases numbered over 1,500, so that his experience entitles him to generalise on the subject. In the whole series he found only four who had absolutely no milk at all, and only six whose inverted nipples proved obstacles which could not be circumvented by care and regular attention.

### Breast Feeding in Doctors' Families.

A still newer contribution to the same question appears in the *American Journal of Obstetrics and Gynæcology* from J. P. Sedgwick, of Minneapolis. This observer decided to find out what the wives of medical men themselves accomplish in the way of nursing their babies. Ignorance can, of course, be put out of count among this class; poverty, one may hope, is not so pressing as to interfere with lactation; and luxury, as a general rule, is certainly not the lot of any large proportion of physicians' families.

In his own State Sedgwick had replies to his circular from 892 doctors, only two of whom had failed to secure some maternal nursing for their offspring; 63 per cent. of the mothers had nursed the full nine months; 10 per cent. more had nursed for six to eight months; 9 per cent. for three to six months; and 16 per cent. for less than three months. In North and South Dakota, Montana, and Northern Iowa the results were even better; here 76 per cent. nursed for nine months. Sedgwick concludes that it is safe to say that 80 per cent. of doctors' wives nurse for three months or more. And he goes on to consider how this percentage and the much lower percentage prevailing in the general population may be increased. The general outline of his recommendations need not be recapitulated here; it agrees with the current teachings of British authorities in essentials. But there are some of his opinions and methods which are rather less familiar, at any rate to general practitioners.

### Some Less Familiar Methods.

Thus he emphasises strongly the futility of depriving the mother of certain articles of diet, such as pickles, sour dishes, etc. Here we think he goes rather too far; there can be no doubt that some mothers do find, after dietary indiscretions of their own, that their babies vomit or display other signs of digestive disturbance. To neglect such indications is merely foolish, and probably the author does not really do so, though his language implies that he considers them of no account.

Contagious diseases, too, according to this writer, are not sufficient grounds for weaning young babies. Scarlet fever, diphtheria, and typhoid are given as instances. Here, again, opinion in Great Britain is not likely to accept such revolutionary ideas. Tuberculosis, however, is admitted to be a valid reason for weaning.

Galactogogues Found Wanting.

Poor quality he will not accept as a proper excuse. Even if a thorough analysis has been conducted by an expert he will not allow that it shows anything material. Poor quality alone he regards as no explanation of failure to gain weight by the infant; quantity, on the other hand, he does attach importance to. Specific galactogogues he does not believe in; the regular suction of a strong infant is, in his opinion, the only good stimulant to lactation. It does not appear whether he has tried the latest galactogogue described in these columns a short time ago (The Hospital, November 16, 1912, p. 200). He takes, however, so thorough and practical an interest in the whole subject of lactation that an independent test by him of the claims made for the substance referred to would be of great value. With his statement that every expectant-mother should be told of the infinitesimal proportion of those in whom lactation is a dead failure when it is properly prepared for and energetically attempted a universal agreement will be felt, and one could wish that investigations similar to this were more abundantly pursued by British investigators.

